# Supplementary material for: Development of a Novel Bronchodilator Vaping Drug Delivery System Based on Thermal Degradation Properties
Source: Pharmaceuticals (Basel). 2023 Dec 15;16(12):1730. doi: 10.3390/ph16121730 (PMC10747077; doi:10.3390/ph16121730)
Supplement: Supplementary file 1 [file pharmaceuticals-16-01730-s001.zip › pharmaceuticals-2686649-supplementary.pdf]

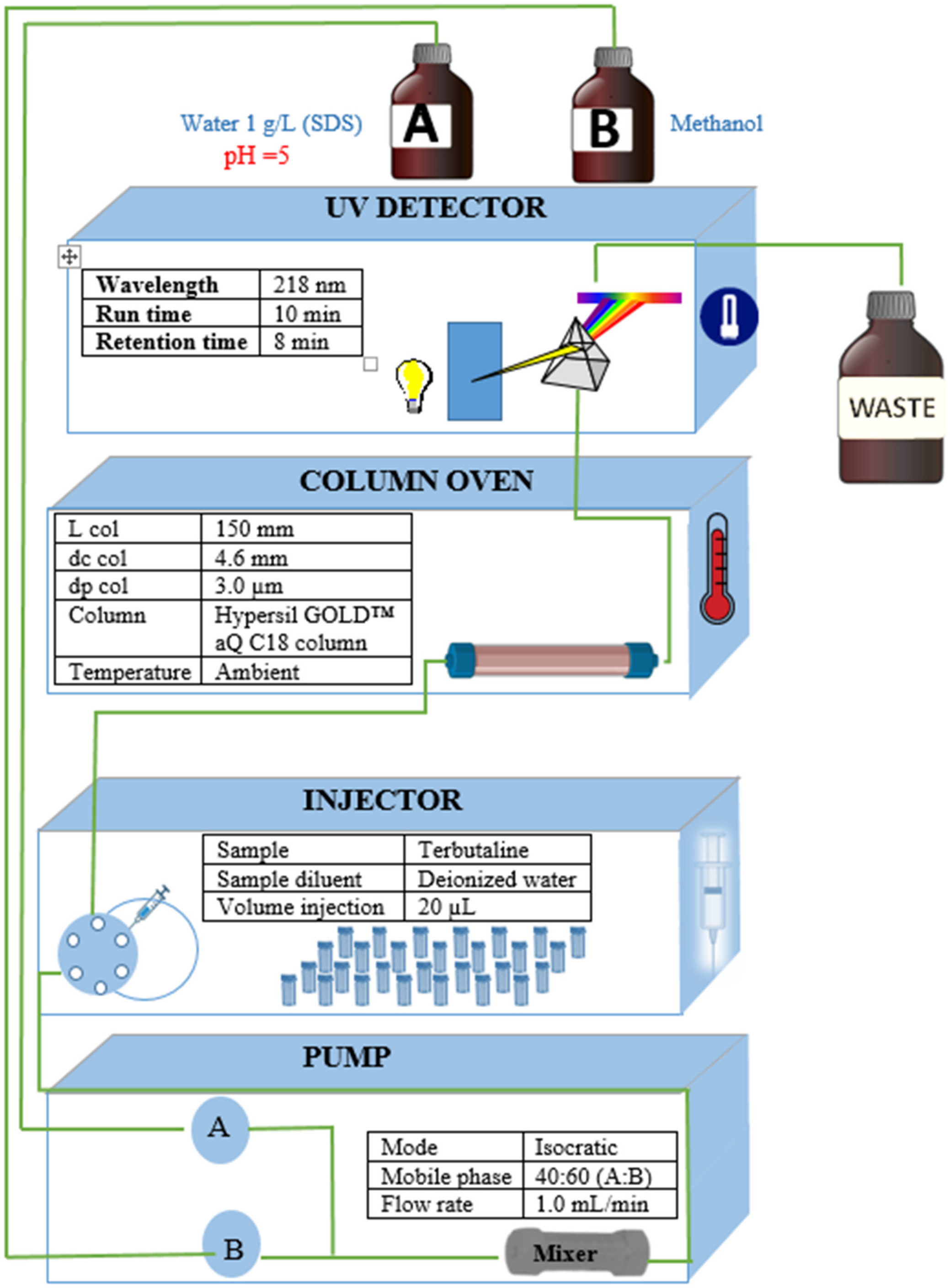

Supplementary material Appendix 1, Figure S1- Chromatographic conditions for Terbutaline quantification.

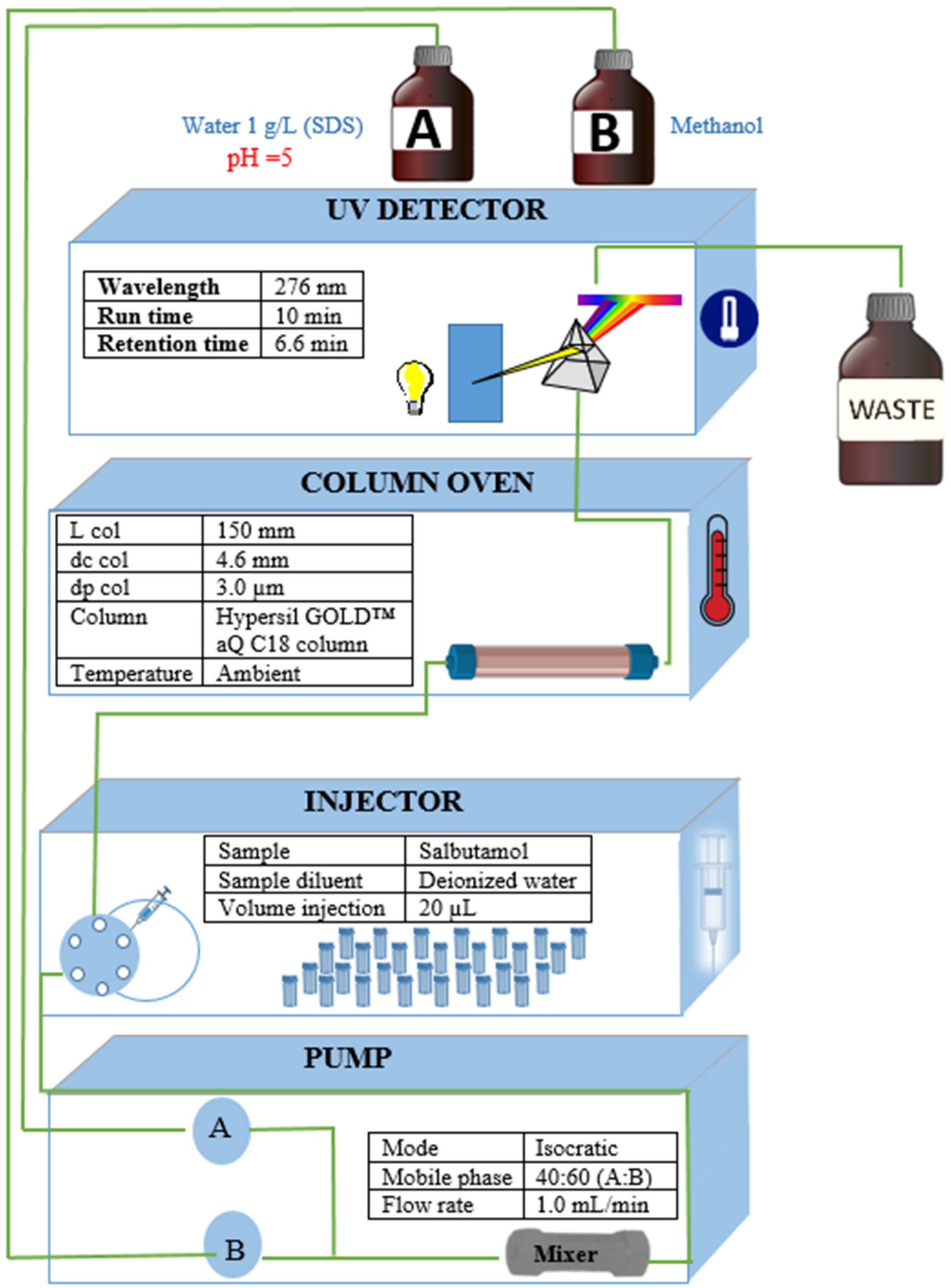

Supplementary material Appendix 1, Figure S2- Chromatographic conditions for Salbutamol hemisulfate quantification.

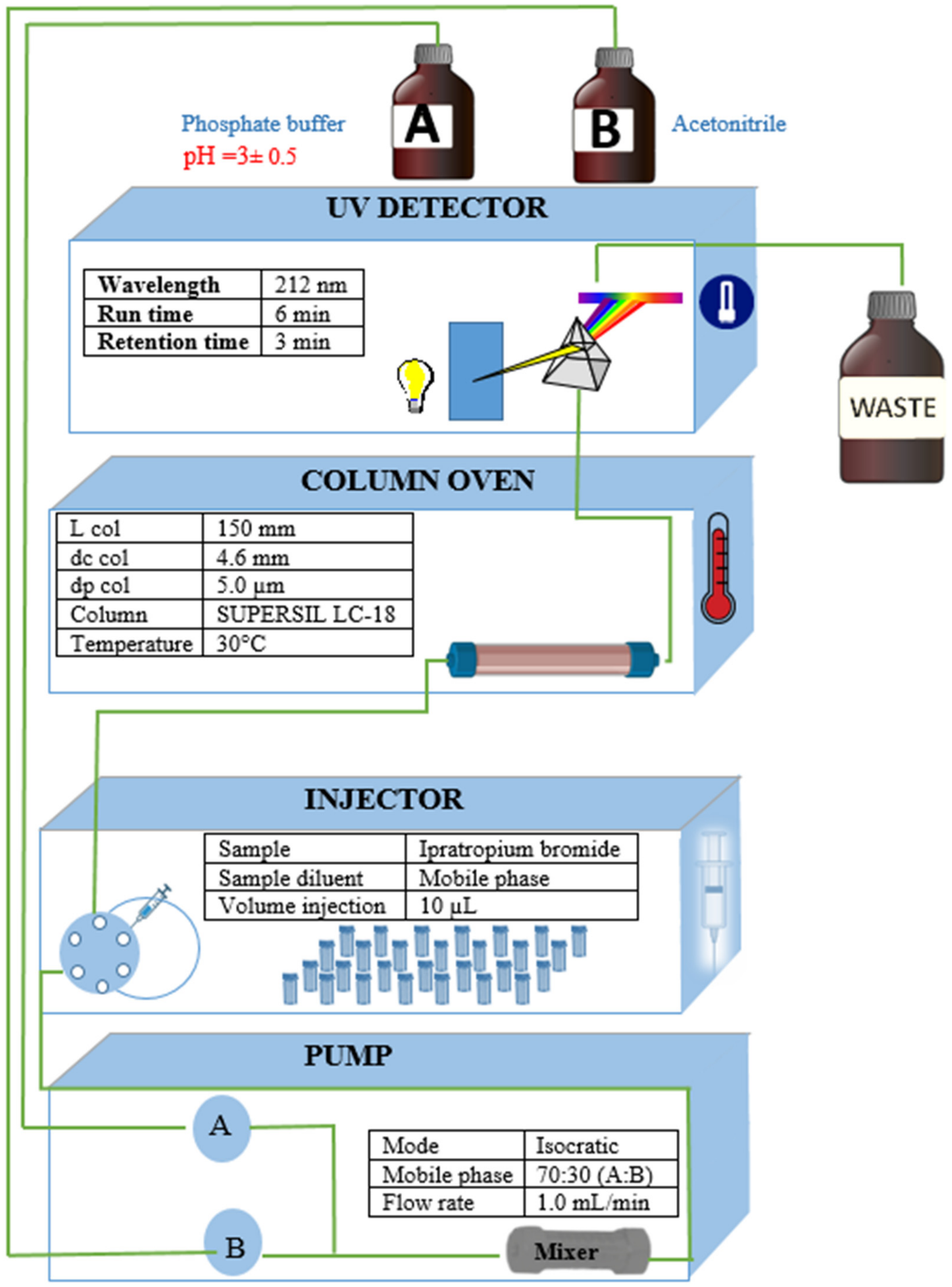

Supplementary material Appendix 1, Figure S3- Chromatographic conditions for Ipratropium bromide quantification.

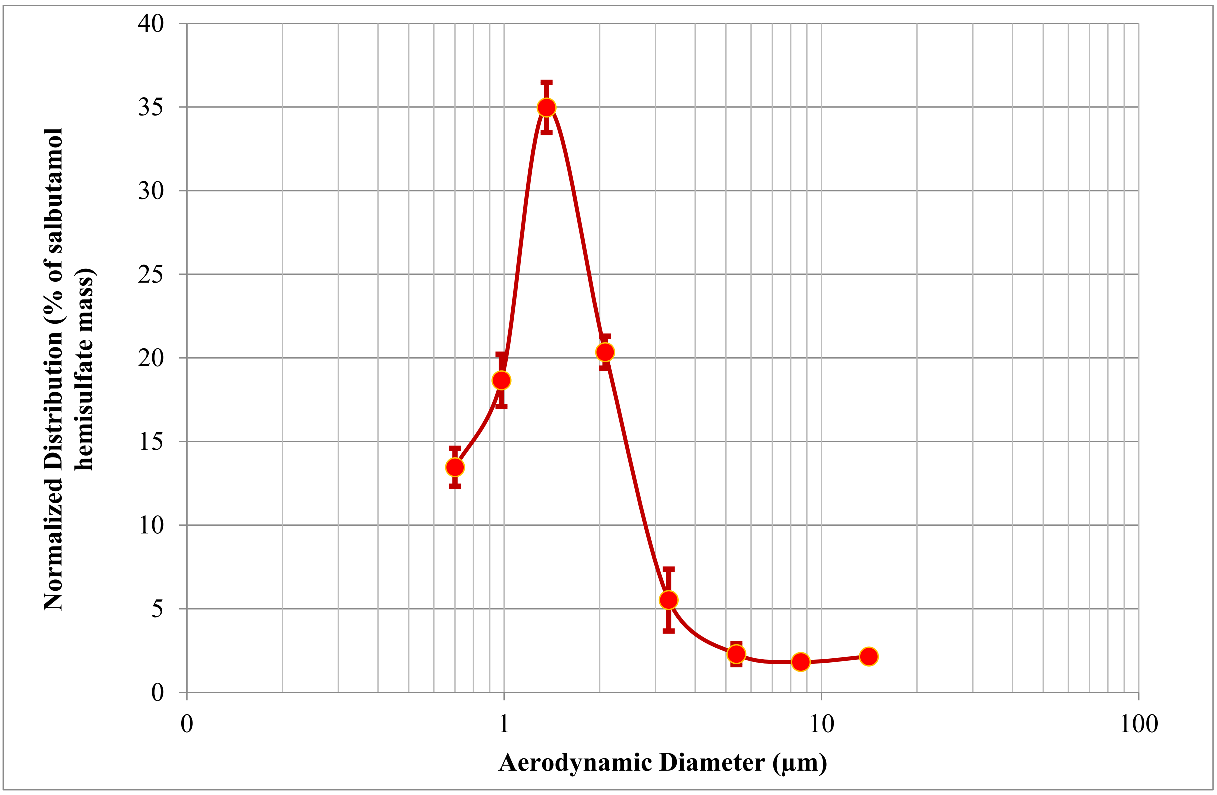

Supplementary material Appendix 2, Figure S1. NGI (Next Generation Impactor) Impactor-collected data showing the MMAD (mass median aerodynamic diameter) of a jet nebulizer (cirrus 2 TM). Results are expressed in µg of salbutamol hemisulfate (mean ± SD, n = 3).

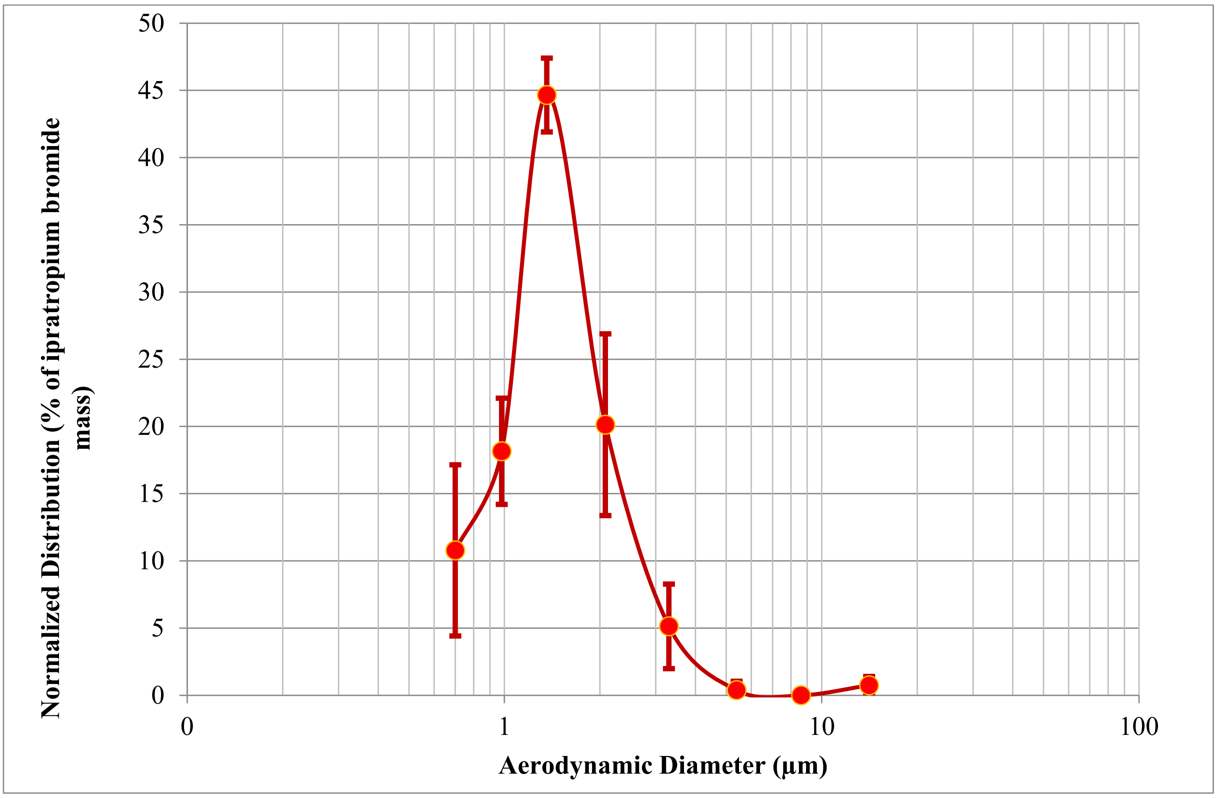

Supplementary material Appendix 2, Figure S2. NGI (Next Generation Impactor) Impactor-collected data showing the MMAD (mass median aerodynamic diameter) of a jet nebulizer (cirrus 2 TM). Results are expressed in µg of ipratropium bromide (mean ± SD, n = 3).

Supplementary data Appendix 3-Validation protocol for the quantification of salbutamol hemisulfate

- Detection method

The method determines salbutamol hemisulfate (bulk form) dissolved in 0.9% NaCl. Samples are analyzed using a Shimadzu HPLC system (Shimadzu, Tokyo, Japan).

- Calibration of standard solutions

Calibration solutions were obtained by dilution of 20mg of salbutamol hemisulfate in 10mL of 0.9% NaCl. Thus, a stock solution of [C] = 2000µg/mL was obtained. Standard solutions were then obtained by further dilution.

Calibration levels of salbutamol hemisulfate are shown below:

**Cal 1 (µg/mL)**	**Cal 2(µg/mL)**	**Cal 3(µg/mL)**	**Cal 4(µg/mL)**	**Cal 5(µg/mL)**	**Cal 6(µg/mL)**
0	600	800	1000	1200	1400

Table 1 Calibration levels for salbutamol hemisulfate.

N.B: Five calibration points are enough to validate the linearity parameter. A [C] = 0µg/mL is mainly used for comparison with higher concentrations to predict the exact retention time and peak of the desired molecule.

- Linearity

Linearity was evaluated in the range of 0 to 1400µg/mL for salbutamol hemisulfate (bulk form).

Linear relationship was observed in the plot of area (mAU.s) as a function of concentration (µg/mL).

Before launching any calibration vial, we used a "front" calibration solution in order to ensure the accuracy of the values that will be obtained on the next vials.

Figure S1 shows the calibration curve obtained via this method.

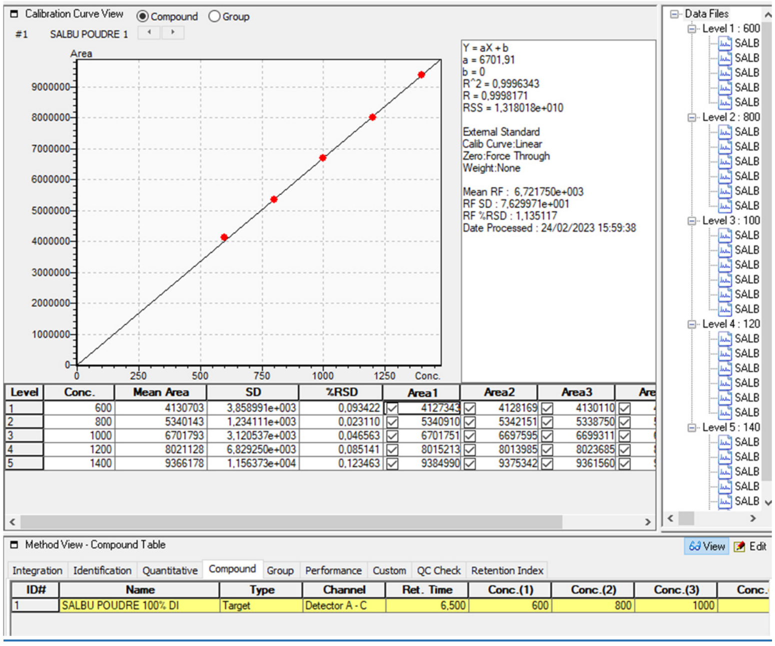

The results obtained for linearity, fully comply with the acceptance criteria set by the ICH guidelines, as the calibration curve has a linear regression coefficient of determination with $R^2 > 0.999$.

- <u>Matrix effect</u>

The matrix effect refers to the interference caused by components of a sample matrix on the measurement of the analyte of interest in an analytical method.

Since we are using e-liquid in our formulation (1, 3-propanediol), it is important to understand and characterize matrix effect to ensure the accuracy and precision of the method.

Consequently, to evaluate the matrix effect, it is essential to take into account the equations assigned in each of the calibration curves (graph below).

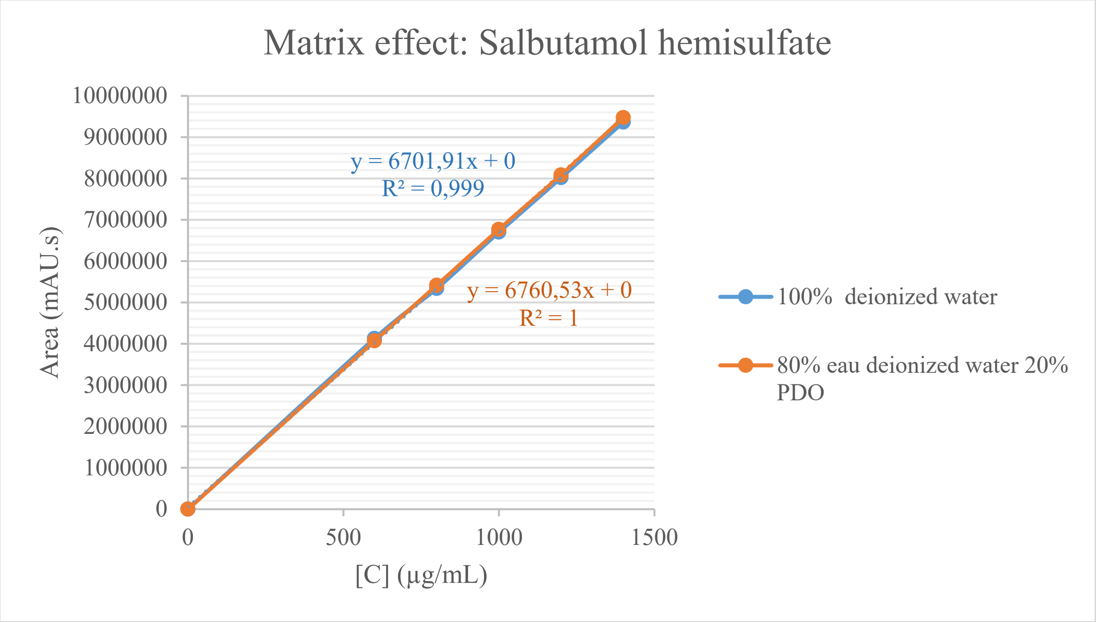

Through these data, we calculate the relative difference of the slopes for each solvent in order to determine the presence or the absence of the matrix effect:

- Salbutamol hemisulfate in: 100% deionized water: 6701.91 X + 0

- Salbutamol hemisulfate in: 20% (1,3-propanediol), 80% deionized water: 6760.53 X + 0

The relative difference of the slopes for each solvent is calculated according to the following formula:

Relative difference= [(Solvent slope in matrix – Solvent slope without matrix) / Solvent slope in matrix] *100

This relative difference must be less than 10% in order to consider the matrix effect as acceptable.

Relative difference= [(6760.53- 6701.91) / 6760.53 *100)] = 0.86% < 10%

- ➢ **Conclusion: absence of matrix effect with the combination of 1,3 propanediol (PDO) and salbutamol hemisulfate.**

- Limit of detection and quantification

Limit of detection (LOD) and limit of quantification (LOQ) are two important concepts in analytical chemistry that relate to the sensitivity of an analytical method.

The limit of detection (LOD) is the lowest amount of an analyte in a sample that can be detected.

The limit of quantification (LOQ) is the lowest amount of an analyte in a sample that can be quantified.

These two parameters are based on the standard deviation of the response (σ) and the slope (S) of the calibration curve

LOD equation

$$LOD = 3.3 \times \frac{\sigma}{S}$$

LOQ equation

$$LOQ = 10 \times \frac{\sigma}{S}$$

Where S is the slope of the calibration line, σ is the residual standard deviation of the regression line. The obtained values of: LOD and LOQ were 2.60 and 7.90 µg/mL, respectively.

[C]	Moyenne des aires (mAU.s)
0	0
600	4070388
800	5415923
1000	6767575
1200	8086961
1400	9471402

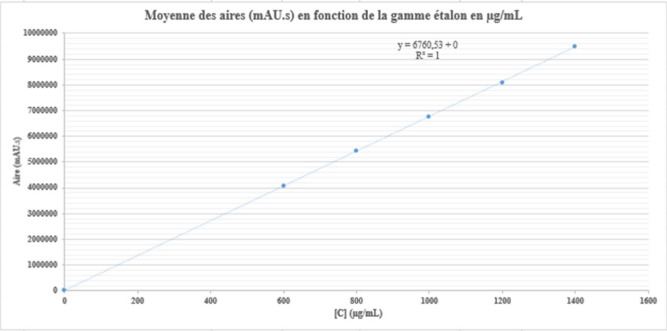

Écart type de la pente	13096,38
Écart type de l'ordonnée à l'origine	5346,574746
LOD	2,609809684
LOQ	7,908514194

N	6
a	6760,53
b	0

RAPPORT DÉTAILLÉ

Statistiques de la régression	
Coefficient de détermination multipl	0,999991645
Coefficient de détermination R^2	0,99998329
Coefficient de détermination R^2	0,999979112
Erreur-type	15330,9969
Observations	6

ANALYSE DE VARIANCE

	Degré de liberté	*Somme des carrés*	*yenne des car*	*F*	*leur critique de F*
Régression	1	5,62611E+13	5,6261E+13	239368,773	1,0471E-10
Résidus	4	940157863,5	235039466		
Total	5	5,6262E+13			

	Coefficients	*Erreur-type*	*Statistique t*	*Probabilité*	*pour seuil de*	*mite supérieure pour seuil de confiance = 9*	*pour seuil de co*	*pour seuil de confiance = 95,0%*
Constante	7004,540541	**13096,38446**	0,53484537	0,62110452	-29356,852	43365,93307	-29356,85199	43365,93307
Variable X 1	6754,044351	13,80480133	489,253281	1,0471E-10	6715,71608	6792,372624	6715,716078	6792,372624

**LOD**	**2.60 µg/mL**
**LOQ**	**7.90 µg/mL**

- Repeatability

Repeatability refers to the degree of agreement between multiple measurements of the same sample, under the same conditions, using the same analytical method and equipment. In other words, it measures the precision of the analytical method.

For this specific reason. the quantification was carried out on two different solutions with the same concentration ([C] =1000µg/mL).

N.B: the acquisition were launched during the same day, by the same operator and under similar experimental conditions.

Each solution was injected 6 times (as mentioned on the table below):

							Mean (µg/mL)
Solution 1	1004.729	1002.35	996.246	997.6	998.97	995.530	999.2375
Solution 2	998.75	999.79	1001.16	1001.14	1003.80	995.53	1000.0283

The given results make it possible to validate the repeatability parameter, as the obtained values are equal to 1000µg/mL.

- Recovery

It involves spiking a known amount of an analyte into a sample, and analyzing the sample to determine the amount of analyte that is recovered after the sample preparation.

As a matter of fact, the recovery is calculated by comparing the measured amount of analyte in the spiked sample to the amount that would be expected to be present based on the known amount of analyte that was added.

This parameter is useful for determining the accuracy and precision of a sample preparation method and can help to ensure that the results obtained from an analytical method are reliable and reproducible.

The theoretical dosing concentration of salbutamol hemisulfate (2 mg/mL)

Group	Theoretical concentration (µg/mL)	Concentration found (µg/mL)	Recovery (%)
60%	600	602	100.33
		600	100
		601	100.16
80%	800	801	100.125
		799	99.875
		802	100.25
100%	1000	1002.349	100.2349
		996.246	99.6246
		997.598	99.7598
120%	1200	1200	100
		1191	99.25
		1200	100
140%	1400	1401.447	100.103357
		1400	100
		1400	100
**Mean % =99.97**			

Table 2: Recovery validation parameter.

Supplementary data Appendix 4-Validation protocol for the quantification of ipratropium bromide

- Detection method

The method determines ipratropium bromide (bulk form) dissolved in 0.9% NaCl. Samples are analyzed using a Shimadzu HPLC system (Shimadzu. Tokyo. Japan).

- Calibration of standard solutions

Calibration solutions were obtained by dilution of 10mg of ipratropium bromide in 10mL of 0.9% NaCl. Thus, a stock solution of [C] = 1000µg/mL was obtained. Standard solutions were then obtained by further dilution.

Calibration levels of ipratropium bromide are shown below:

**Cal 1(µg/mL)**	**Cal 2(µg/mL)**	**Cal 3(µg/mL)**	**Cal 4(µg/mL)**	**Cal 5(µg/mL)**
**75**	100	125	150	175

Table 2 Calibration levels for salbutamol hemisulfate.

- Linearity

Linearity was evaluated in the range of 75 to 175µg/mL for ipratropium bromide (bulk form).

Linear relationship was observed in the plot of area (mAU.s) as a function of concentration (µg/mL).

Before launching any calibration vial, we used a “front” calibration solution in order to ensure the accuracy of the values that will be obtained on the next vials.

Figure S1 shows the calibration curve obtained via this method.

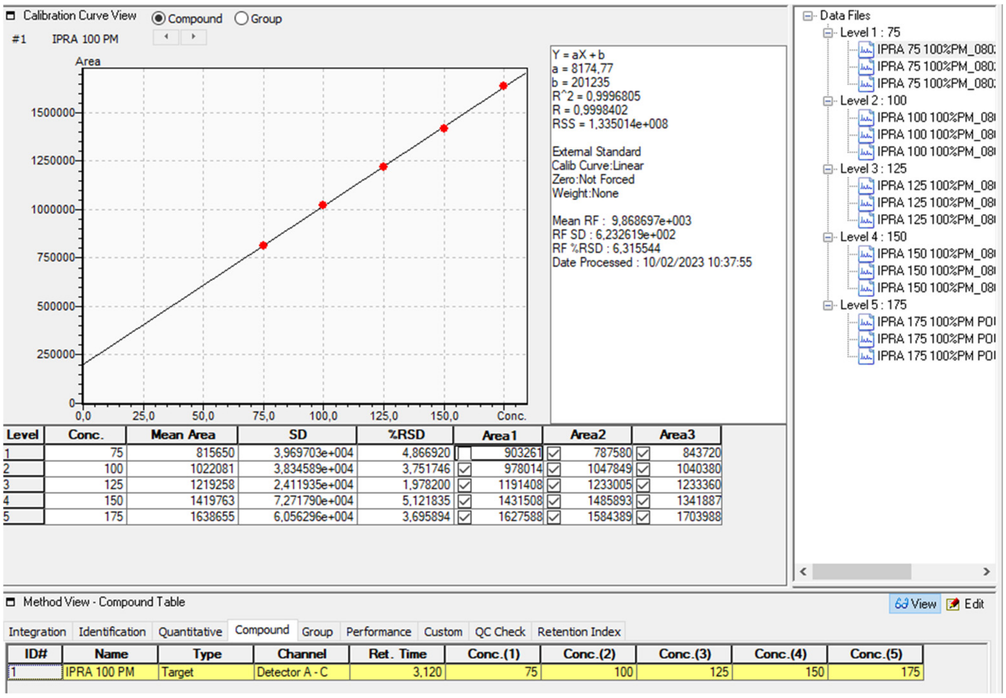

Level	Conc.	Mean Area	SD	%RSD	Area1	Area2	Area3
1	75	815650	3,969703e+004	4,866920	903261	787580	843720
2	100	1022081	3,834589e+004	3,751746	978014	1047849	1040380
3	125	1219258	2,411935e+004	1,978200	1191408	1233005	1233360
4	150	1419763	7,271790e+004	5,121835	1431508	1485893	1341887
5	175	1638655	6,056296e+004	3,695894	1627588	1584389	1703988

ID#	Name	Type	Channel	Ret. Time	Conc.(1)	Conc.(2)	Conc.(3)	Conc.(4)	Conc.(5)
1	IPRA 100 PM	Target	Detector A - C	3,120	75	100	125	150	175

The results obtained for linearity, fully comply with the acceptance criteria set by the ICH guidelines, as the calibration curve has a linear regression coefficient of determination with R^2 > 0.999.

- <u>Matrix effect</u>

The matrix effect refers to the interference caused by components of a sample matrix on the measurement of the analyte of interest in an analytical method.

Since we are using e-liquid in our formulation (1, 3-propanediol), it is important to understand and characterize matrix effect to ensure the accuracy and precision of the method.

Consequently, to evaluate the matrix effect, it is essential to take into account the equations assigned in each of the calibration curves (graph below).

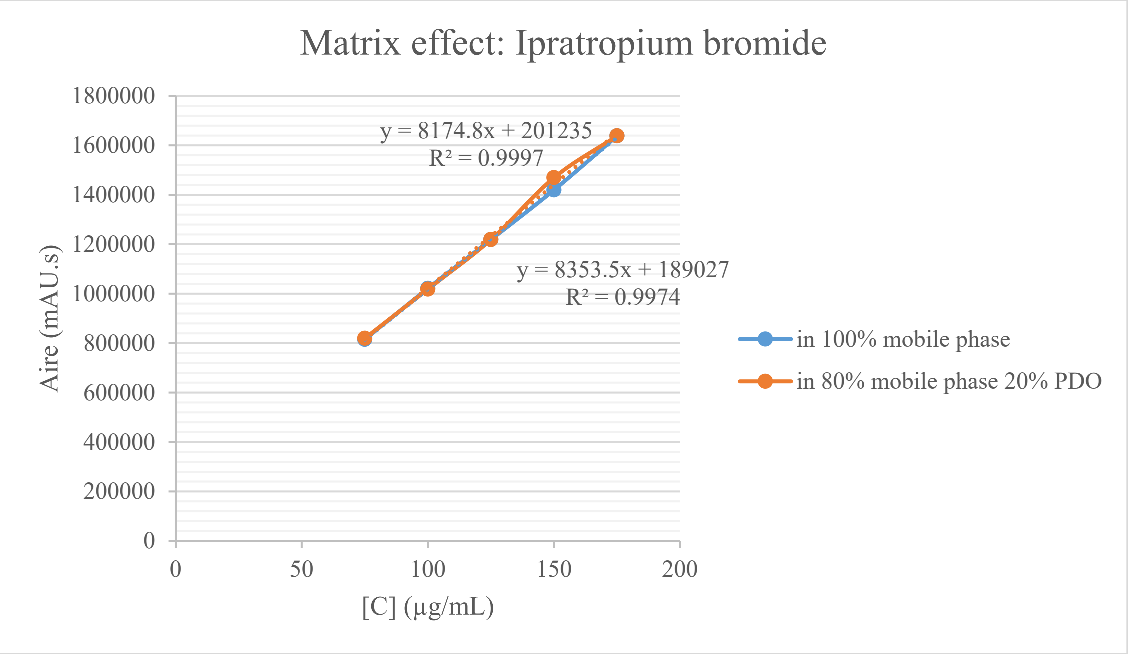

Through these data, we calculate the relative difference of the slopes for each solvent in order to determine the presence or the absence of the matrix effect:

- Ipratropium bromide in: 100% mobile phase (30% acetonitrile: 70% phosphate buffer): 8174.8 X + 201235

- Ipratropium bromide in: 20% (1,3-propanediol), 80% deionized water: 8353.5 X + 189027

The relative difference of the slopes for each solvent is calculated according to the following formula:

Relative difference= [(Solvent slope in matrix – Solvent slope without matrix) / Solvent slope in matrix] *100

This relative difference must be less than 10% in order to consider the matrix effect as acceptable.

Relative difference= [(8353.5- 8174.8) / 8353.5 *100)] = 2.13 < 10%

- **Conclusion: absence of matrix effect with the combination of 1,3 propanediol (PDO) and ipratropium bromide.**

- <u>Limit of detection and quantification</u>

Limit of detection (LOD) and limit of quantification (LOQ) are two important concepts in analytical chemistry that relate to the sensitivity of an analytical method.

The limit of detection (LOD) is the lowest amount of an analyte in a sample that can be detected.

The limit of quantification (LOQ) is the lowest amount of an analyte in a sample that can be quantified.

These two parameters are based on the standard deviation of the response (σ) and the slope (S) of the calibration curve

LOD equation

$$LOD = 3.3 \times \frac{\sigma}{S}$$

LOQ equation

$$LOQ = 10 \times \frac{\sigma}{S}$$

Where S is the slope of the calibration line, σ is the residual standard deviation of the regression line. The obtained values of: LOD and LOQ were 5.70 and 17.28µg/mL, respectively.

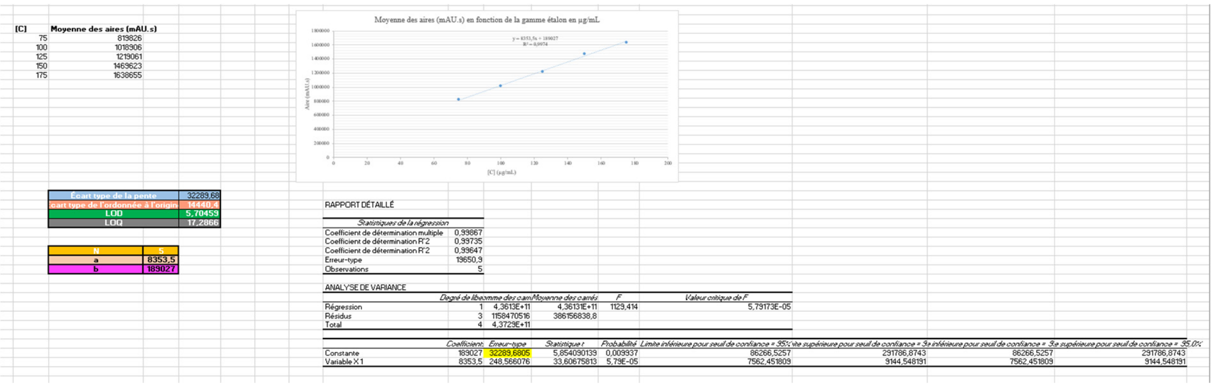

[C]	Moyenne des aires (mAU.s)
75	819826
100	1018906
125	1219061
150	1469623
175	1638655

Écart type de la pente	32289,68
cart type de l'ordonnée à l'origin	14440,4
LOD	5,70459
LOQ	17,2866

N	5
a	8353,5
b	189027

RAPPORT DÉTAILLÉ

Statistiques de la régression	
Coefficient de détermination multiple	0,99867
Coefficient de détermination R^2	0,99735
Coefficient de détermination R^2	0,99647
Erreur-type	19650,9
Observations	5

ANALYSE DE VARIANCE

	Degré de libe	*omme des carr*	*Moyenne des carrés*	*F*	*Valeur critique de F*
Régression	1	4,3613E+11	4,36131E+11	1129,414	5,79173E-05
Résidus	3	1158470516	386156838,8		
Total	4	4,3729E+11			

	Coefficient	*Erreur-type*	*Statistique t*	*Probabilité*	*Limite inférieure pour seuil de confiance = 95%*	*ite supérieure pour seuil de confiance = 9*	*e inférieure pour seuil de confiance = 9*	*e supérieure pour seuil de confiance = 95,0%*
Constante	189027	32289,6805	5,854090139	0,009937	86266,5257	291786,8743	86266,5257	291786,8743
Variable X 1	8353,5	248,566076	33,60675813	5,79E-05	7562,451809	9144,548191	7562,451809	9144,548191

**LOD**	**5.70 µg/mL**
**LOQ**	**17.28 µg/mL**

- <u>Repeatability</u>

Repeatability refers to the degree of agreement between multiple measurements of the same sample, under the same conditions, using the same analytical method and equipment. In other words, it measures the precision of the analytical method.

For this specific reason. the quantification was carried out on two different solutions with the same concentration ([C] =125µg/mL).

N.B: the acquisition were launched during the same day, by the same operator and under similar experimental conditions.

Each solution was injected 6 times (as mentioned on the table below):

							**Mean (µg/mL)**
Solution 1	125.20	125.00	125.1	125.40	125.06	124.80	125.09
Solution 2	124.95	124.94	125.01	125.13	125.02	124.98	125.009

The given results make it possible to validate the repeatability parameter, as the obtained values are equal to 125µg/mL.

- <u>Recovery</u>

It involves spiking a known amount of an analyte into a sample, and analyzing the sample to determine the amount of analyte that is recovered after the sample preparation.

As a matter of fact, the recovery is calculated by comparing the measured amount of analyte in the spiked sample to the amount that would be expected to be present based on the known amount of analyte that was added.

This parameter is useful for determining the accuracy and precision of a sample preparation method and can help to ensure that the results obtained from an analytical method are reliable and reproducible.

The theoretical dosing concentration of ipratropium bromide (250 µg/mL)

Group	Concentration theoretical (µg/mL)	Concentration found (µg/mL)	Recovery (%)
60%	75	73.312	97.7493333
	75	77.310	103.08
	75	76.918	102.557333
80%	100	100.503	100.503
	100	98.588	98.588
	100	98.974	98.974
100%	125	119.926	95.9408
	125	120.173	96.1384
	125	129.819	103.8552
120%	150	146.447	97.6313333
	150	156.55	104.366667
	150	156.905	104.603333
140%	175	173.54	99.02857143
	175	170.602	97.48685714
	175	180.1	102.9142857
**Moyenne% =100.22**			

Table 2: Recovery validation parameter.
